# Special Issue “From Genes to Medicine: Exploring the Genetic and Biological Bases of Medicinal Plants”

**DOI:** 10.3390/ijms27219685

**Published:** 2026-10-29

**Authors:** Wajid Zaman

**Affiliations:** Department of Life Sciences, Yeungnam University, Gyeongsan 38541, Republic of Korea; wajidzaman@yu.ac.kr

## 1. Introduction

Medicinal plants are chemically heterogeneous biological systems: their chemical and ecological phenotypes emerge from the interaction of lineage-specific genomic variation, tissue- and developmental regulation, enzymatic pathway architecture, and environment. For translational work, a candidate metabolite requires more than detection in an extract; its identity, quantitative range, biosynthetic origin, and biological activity must be connected to a reproducible source genotype and production context. Recent genomic resources have expanded the ability to identify biosynthetic genes and compare pathway evolution [1], while integrated omics increasingly resolve gene, protein, and metabolite relationships [2]. An association between transcript abundance and chemical abundance still requires causal testing, and observed pharmacological activity requires assessment of exposure, selectivity, and safety before clinical relevance can be inferred. The five contributions in this Special Issue span sequence-based authentication, stress-response and defense analyses, and a synthesis of genome-guided biotechnology. Read together, they support a mechanistic account of translation in which genetic identity and pathway activity are interpreted alongside environmental responses and analytical quality. This evidence chain clarifies what current molecular data can establish and identifies the experimental transitions that remain unresolved.

## 2. Genomic Variation and Biosynthetic Mechanisms

**Figure 1:**
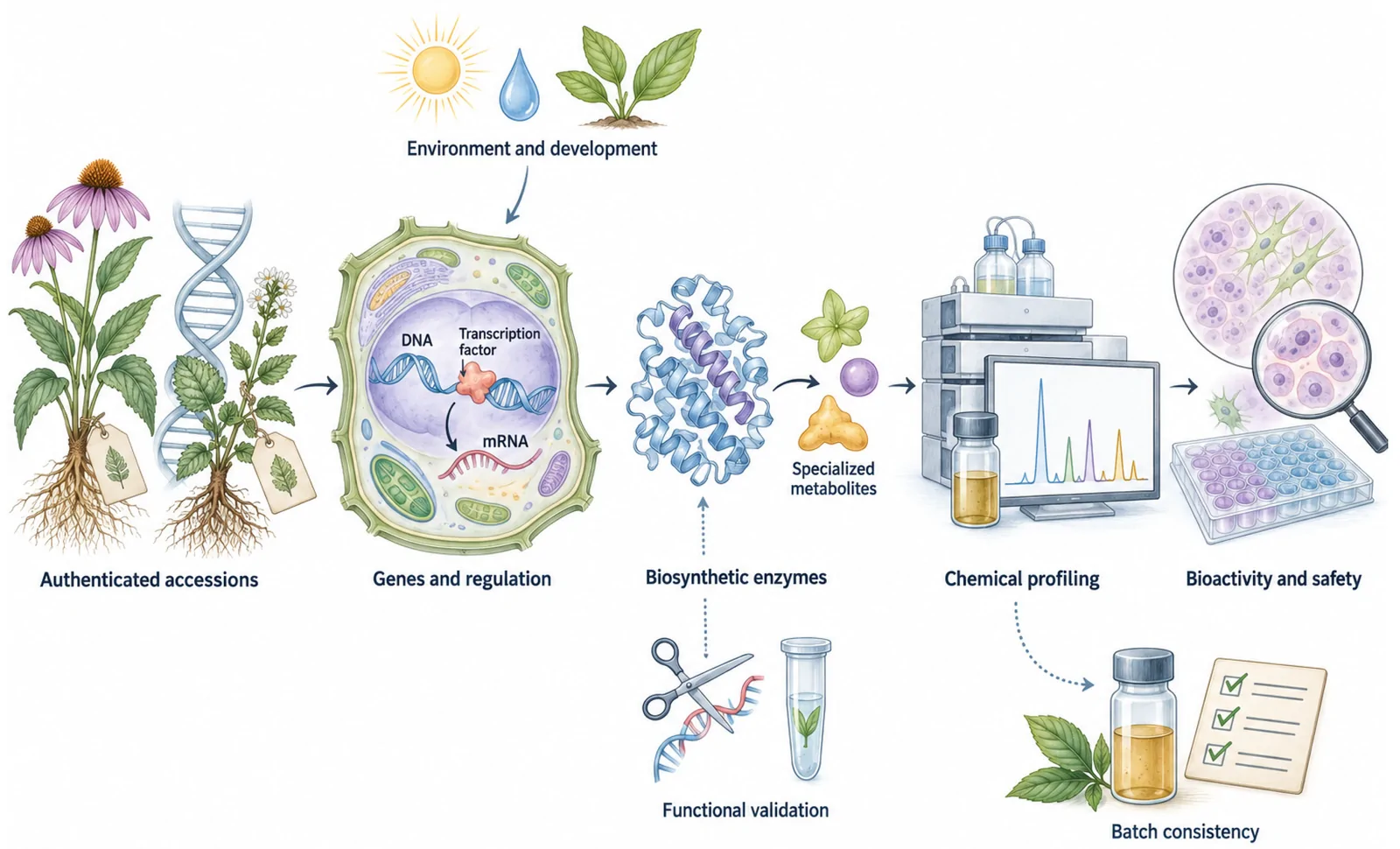
Conceptual route linking medicinal-plant genetic diversity to chemical characterization and product quality. The workflow integrates sequence, expression, pathway validation, analytical, and bioactivity evidence; it does not imply that any single accession or extract has established clinical efficacy. Dotted links denote supporting validation or quality-control relationships; the chromatogram is schematic.

High-quality genome assemblies establish the sequence framework for identifying pathway genes, duplicated families, and candidate regulatory regions [1]. Comparative genomics then places those candidates in an evolutionary context, while transcriptomics, proteomics, and metabolomics can test whether predicted enzymes and regulators track with tissue-specific chemical phenotypes [2]. These layers are complementary: genomic association nominates candidates, expression patterns prioritize states and tissues, and biochemical measurements determine whether the predicted product accumulates. Functional perturbation is needed to establish which candidates control flux through a pathway. The elucidation of missing vinblastine biosynthetic steps illustrates the need to verify candidate enzyme activities experimentally [3]. The review by Zaman and Park (contribution 1) surveys RNA sequencing, CRISPR/Cas9 editing, synthetic biology, metabolic engineering, and culture-based production, framing how discovery can progress toward scalable output while recognizing validation and production constraints. Production of artemisinic acid in engineered yeast provides an example of reconstructing plant biosynthetic functions in a heterologous host [4]. In medicinal plants, this sequence also has a conservation dimension because genetic diversity provides a resource for biosynthetic discovery and breeding [1]. Figure 1 summarizes the evidence path from population variation through pathway regulation and chemical profiling to bioactivity and safety assessment and evaluation of batch consistency.

## 3. Authentication and Botanical Quality

Botanical identity is an initial quality-control condition for medicinal use because substitution changes the genetic and chemical material under evaluation. A synthesis of DNA-based surveys covering 5957 commercial herbal products from 37 countries found that 27% were adulterated relative to their labeled species [5]. DNA barcoding can support species discrimination in traded material, although closely related taxa, incomplete reference libraries, and degraded samples can limit resolution; the Lamiaceae review documents these methodological constraints and the need to choose markers in relation to the taxa and material being tested [6]. Within the Special Issue, Chouksey and colleagues (contribution 2) developed a one-tube multiplex assay using sequence-characterized amplified region markers derived from RAPD and ISSR profiles. The assay distinguished *Senna alexandrina* from *Cassia fistula* and *Senna sophera*, with multiplex results concordant with corresponding single-target assays. This provides a targeted authentication route for the tested species and sample set. It does not by itself quantify sennosides or characterize other chemical constituents, so molecular identification and chemical profiling serve complementary purposes in botanical quality assessment.

## 4. Environmental Response and Functional Evidence

**Figure 2:**
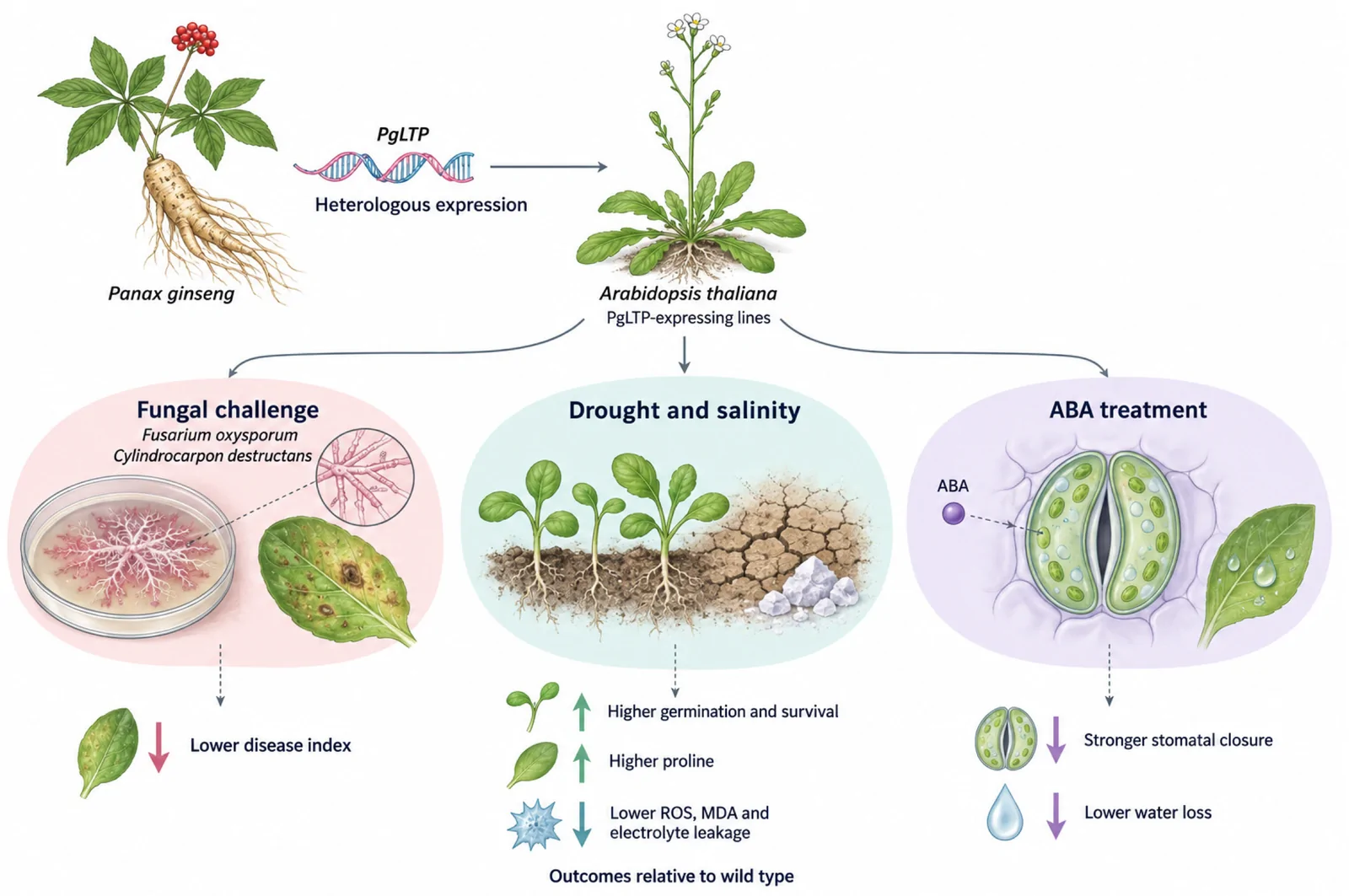
Stress-associated phenotypes reported after heterologous expression of PgLTP in *Arabidopsis thaliana* (contribution 3). The study measured disease index, germination and survival under stress, proline, oxidative damage, electrolyte leakage, and ABA-responsive stomatal behavior. Dotted links indicate associated physiological readouts; the complete signaling pathway and activity in *Panax ginseng* remain unestablished. ABA, abscisic acid; ROS, reactive oxygen species; MDA, malondialdehyde.

Environmental conditions modulate the same gene-to-metabolite relationships that define medicinal-plant traits. Water limitation, salinity, pathogens, developmental stage, and harvest timing can alter gene expression, redox status, biomass allocation, and specialized-metabolite abundance, making a genotype-to-product relationship conditional on the growth environment [2]. The ginseng study in this collection (contribution 3) characterized a *Panax ginseng* lipid-transfer-protein candidate, PgLTP, and assessed it through heterologous expression in *Arabidopsis thaliana*. The transgenic lines showed lower disease indices after fungal challenge, improved germination and survival under drought and salinity, increased proline, reduced oxidative damage and electrolyte leakage, and stronger ABA-responsive stomatal closure associated with lower water loss relative to the wild type. These readouts link the candidate gene to several stress-associated phenotypes in a model host. They do not establish the complete signaling sequence or demonstrate the same effects in ginseng. Figure 2 summarizes the reported experimental context and measured outcomes, with physiological readouts presented as associated responses rather than a fully resolved causal pathway.

## 5. Plant Defense Across Biological Scales

The grapevine contribution extends the collection’s molecular perspective to infection-stage resolution. Pandiyan and Park (contribution 4) reanalyzed a public RNA-sequencing dataset for *Vitis vinifera* challenged by *Xylella fastidiosa* and reported 1093 differentially expressed genes in an early phase and 136 in an intermediate phase. Their network analysis highlighted defense-associated candidates, including EDS1, a chitinase, and a thaumatin-like protein. The contrast between phases shows why sampling time is integral to interpreting plant defense: a network inferred from an early response can differ substantially from one measured after symptoms progress. Because the study reanalyzed an existing transcriptome, its hub genes are candidates for functional validation; expression and network centrality alone do not establish that a gene causes resistance. The study therefore contributes a temporal map of host responses and a set of testable hypotheses. Its value for medicinal-plant research lies in the broader experimental principle that pathogen exposure and disease stage can reshape plant molecular states relevant to cultivation and biomass quality.

A further contribution examines the white-backed planthopper, *Sogatella furcifera*, and its response to etofenprox. Khan and colleagues (contribution 5) report plasticity in gene-expression patterns and identify CYPSF (labeled CYP5F in the study figures) as a candidate associated with the resistant population. Piperonyl butoxide increased etofenprox susceptibility, supporting the involvement of P450-mediated detoxification without identifying the responsible isoform. This work concerns an insect response and does not directly measure medicinal-plant biosynthesis, chemotype, or therapeutic activity. Its inclusion broadens the biological frame to pest pressure and detoxification-related expression in plant-associated systems. That distinction matters for synthesis: adjacent evidence can motivate questions about cultivation conditions and biotic interactions, while claims about medicinal-plant traits still require direct measurements in the relevant plant species. The article also illustrates a recurring evidential boundary in transcriptomic work. Expression differences can identify candidate mechanisms, but gene-specific perturbation and biochemical assays are needed to establish the contribution of CYPSF to resistance. Kept at this evidential level, the study complements the plant-pathogen analysis by showing how molecular responses can be dynamic across interacting organisms.

## 6. Integrating Molecular Evidence with Translation

Together, the collection links three questions that are often considered separately: what biological material is present, how its molecular networks respond to genotype and environment, and whether those responses produce a stable chemical phenotype. A stronger gene-to-medicine evidence chain would connect authenticated accessions to replicated, multi-environment phenotyping; quantify candidate metabolites with validated analytical methods; and test prioritized enzymes or regulators through perturbation, rescue, or heterologous reconstruction. Fermentation of artemisinic acid followed by chemical conversion to artemisinin illustrates the additional processing needed beyond pathway reconstruction [7]. The resulting compounds or standardized extracts would then require biological assays that report concentration, target engagement, selectivity, and toxicity. This framework also clarifies conservation priorities: genetic resources merit protection when distinct populations contain verified biosynthetic or adaptive variation, and cultivation can be evaluated for both yield and chemical consistency [2]. The present contributions provide pieces of this chain, from a practical Senna identity assay to stress-associated functional evidence and a review of biotechnology options. Their combined implication is that medicinal-plant translation depends on linking sequence, function, chemistry, and biological effect while retaining the limits of each evidence type.

## Data Availability

No new data were generated or analyzed in this editorial.
